# Evaluation of potential biomarkers during irinotecan-based systemic treatment for colorectal cancer—study protocol of the OPTIMA study

**DOI:** 10.1186/s12885-025-14500-6

**Published:** 2025-07-01

**Authors:** E. Russ, J. Ziemons, L. E. Hillege, S. M. J. van Kuijk, E. M. de Jong, C. Elbers, M. J. Deenen, L. H. Borghuis, T. M. M. Böhm, P. Kristen, L. C. Valk, I. E. G. van Hellemond, H. Vestjens, A. Dietvorst, A. Baars, A. N. M. Goosens, L. Vermeulen, T. E. Buffart, J. de Vos-Geelen, J. Penders, M. R. Redinbo, L. Valkenburg-van Iersel, M. L. Smidt

**Affiliations:** 1 GROW– Research Institute for Oncology and Reproduction, Maastricht University, Maastricht, the Netherlands; 2 Department of Surgery, Maastricht University Medical Center+, Maastricht, the Netherlands; 3 Department of Epidemiology, Faculty of Health, Medicine and Life Sciences, Maastricht University, Maastricht, the Netherlands; 4 Catharina Cancer Institute, Catharina Hospital, Eindhoven, the Netherlands; 5 Amsterdam UMC Location University of Amsterdam, Center for Experimental and Molecular Medicine, Laboratory of Experimental Oncology and Radiobiology, Amsterdam, the Netherlands; 6 Oncode Institute, Amsterdam, the Netherlands; 7 Cancer Center Amsterdam, Cancer Biology, Amsterdam, the Netherlands; 8 Department of Clinical Pharmacy, Catharina Hospital, Eindhoven, the Netherlands; 9 Department of Clinical Pharmacy and Toxicology, Leiden University Medical Center, Leiden, the Netherlands; 10 Wageningen University & Research, Wageningen, the Netherlands; 11 Department of Internal Medicine, Catharina Hospital, Eindhoven, the Netherlands; 12 Department of Internal Medicine, VieCuri, Venlo, the Netherlands; 13 Department of Internal Medicine, Van Weel-Bethesda Hospital, Dirksland, the Netherlands; 14 Department of Internal Medicine, Hospital Gelderse Vallei, Ede, the Netherlands; 15 Departmnet of Internal Medicine, Rode Kruis Hospital, Beverwijk, the Netherlands; 16 Department of Medical Oncology, Amsterdam UMC, Amsterdam, the Netherlands; 17 Department of Internal Medicine, Division of Medical Oncology, Maastricht University Medical Centre+, Maastricht, the Netherlands; 18 NUTRIM– Research Institute of Nutrition and Translational Research In Metabolism, Maastricht University, Maastricht, the Netherlands; 19 Department of Medical Microbiology, Infectious Diseases and Infection Prevention, Maastricht University Medical Centre+, Maastricht, the Netherlands; 20 Departments of Chemistry, Biochemistry & Biophysics, and Microbiology & Immunology, University of North Carolina, Chapel Hill, NC USA

**Keywords:** Colorectal cancer, Irinotecan, Chemotherapy efficacy, Toxicity, Consensus molecular subtypes, UGT1A1, β-glucuronidase, Gut microbiota

## Abstract

**Background:**

Patients with advanced colorectal cancer (CRC) commonly receive irinotecan-based systemic treatment to alleviate symptoms, improve quality of life (QoL), and prolong overall survival (OS). However, predicting efficacy and toxicity of the treatment is challenging. Previous research indicated an association between the tumor molecular profile and response to irinotecan-based systemic treatment. Moreover, the *UGT1A1* genotype of the patient, and the activity of the gut microbial enzyme β-glucuronidase (GUS) have been suggested as biomarkers for the development of systemic (e.g. neutropenia) and gastrointestinal toxicity (e.g. diarrhea) respectively. Therefore, the OPTIMA study will evaluate in patients with advanced CRC: 1) whether tumor molecular profiling can predict the efficacy of irinotecan-based systemic treatment; 2) whether high bacterial GUS enzyme activity is associated with increased gastrointestinal toxicity, decreased QoL and OS; as well as 3) the safety of a 70% irinotecan dose intensity in UGT1A1 poor metabolizers (PMs).

**Methods:**

This prospective, observational, multi-center cohort study will include patients with advanced CRC scheduled for irinotecan-based systemic treatment. Archived tumor tissue and routine CT/MRI scans at baseline and after four cycles will be used to investigate the association of tumor molecular profile and treatment response according to RECIST. Before treatment initiation, germline DNA obtained from whole blood will be genotyped using PCR to determine the UGT1A1*28 genotype, followed by 30% irinotecan dose reduction in UGT1A1 PMs. Bacterial GUS activity will be quantified in fecal samples collected before and during treatment by means of an enzyme activity assay and will be related to patient-reported gastrointestinal toxicity (mainly diarrhea). Additionally, patients will fill in questionnaires concerning QoL, medication use, medical history and comorbidities, dietary habits, as well as physical performance. OS will be documented, capturing the duration from the start of treatment until death from any cause.

**Discussion:**

Results obtained in the context of the OPTIMA study are expected to contribute to the optimization of efficacy and the reduction of toxicity of irinotecan-based systemic treatment, thereby potentially improving QoL of patients. Given that maintaining QoL is particularly critical in the palliative setting, the OPTIMA study has the potential to be of significant benefit for patients and their caregivers.

**Trial registration:**

This trial is registered in the ClinicalTrials.gov register (NCT05655780) on December 16th, 2022.

## Background

Colorectal cancer (CRC) represents a major public health concern as it is the third most frequently diagnosed cancer and the second cause of cancer-related mortality worldwide [1]. In 2022, 21% of patients with newly diagnosed CRC in the Netherlands suffered from metastatic disease [2]. In this group of patients, irinotecan-based systemic treatments are commonly applied to relieve symptoms, improve quality of life (QoL) and extend overall survival (OS).

However, the individual response to irinotecan-based treatment differs consistently between patients, and is currently largely unpredictable. Previous research indicated that molecular tumor characteristics might be associated with response to irinotecan-based systemic treatment. CRC can be subdivided into four consensus molecular subtypes (CMS1-4), based on different gene expression profiles [3]. Ten Hoorn et al. concluded based on a meta-analysis that specifically in CMS4 metastatic disease, patients benefit more from irinotecan-based regimens compared with oxaliplatin-containing regimens [4].

An additional disadvantage of irinotecan-based systemic treatment is the occurrence of systemic (primarily neutropenia) and late-onset gastrointestinal (GI) toxicity, (primarily diarrhea). For instance, grade 3–4 neutropenia has been previously identified in 46% and grade 3–4 diarrhea in approximately 18% of patients [5, 6]. The occurrence of severe toxicity often leads to irinotecan dose reduction, or premature discontinuation of the treatment. This negatively affects not only the patient’s QoL and OS, but also puts significant pressure on family and caregivers, as well as on hospitalization-related healthcare costs [7]. Consequently, there is an urgent need for biomarkers to identify patients at risk for developing irinotecan-induced toxicity. In this context, two promising candidate biomarkers are 1) the UDP Glucuronosyltransferase Family 1 Member A1 (*UGT1A1*) genotype of the patient and 2) the microbial enzyme β-glucuronidase (GUS).

UGT1A1 is the main liver enzyme responsible for inactivation of SN-38 (the active metabolite of irinotecan) by conjugating it with glucuronic acid, resulting in SN-38G [8]. Certain *UGT1A1* polymorphisms (UGT1A1*28 and UGT1A1*93) have been shown to be associated with reduced enzyme activity and an increased risk to develop neutropenia [8–10]. To prevent this toxicity, the clinical utility of *UGT1A1* genotyping with subsequent initial genotype-guided dose adaptation has been demonstrated [8]; however, is not yet routinely recommended in Dutch clinical guidelines [11].

The microbial enzyme GUS is expressed by certain gut bacteria [12]. GUS enzymes can remove glucuronic acid for microbial use as a carbon source, and in this way re-activate the inactive SN-38G into the cytotoxic SN-38 in the intestinal lumen [13]. This reactivation process has significant clinical implications, potentially resulting in severe mucosal damage and diarrhea [14]. Preclinical studies have demonstrated that the inhibition of GUS might be a promising method to limit SN-38-induced GI toxicity [15, 16]. However, this association has not been investigated in humans yet and requires further investigation. In addition, there are hundreds of different GUS enzyme variants found in human stool samples, with a broad diversity in structures, and functionality [12, 17, 18]. Therefore, it also remains to be established which specific microbes exert SN-38G-specific GUS activity and are related to irinotecan-induced GI toxicity in CRC patients.

In view of the studies described above, the tumor molecular profile, *UGT1A1* genotype and bacterial GUS are promising molecular markers for the prediction of irinotecan efficacy and toxicity. Therefore, the OPTIMA study aims to evaluate 1) whether tumor molecular profiling (e.g. CMS classification, transcriptomics) can predict the efficacy of irinotecan-based systemic treatment and is associated with QoL and OS, and 2) whether UGT1A1-guided dose reduction in UGT1A1 poor metabolizers (PMs) is safe to use, and 3) whether high GUS enzyme activity is associated with GI toxicity, decreased QoL and OS in patients with metastatic or unresectable CRC. These insights will greatly contribute to more personalized irinotecan-based treatment regimens as well as well-considered choices for other treatment options.

## Methods and study design

### Aims of the study

The primary objectives of this study are:

1. To investigate whether tumor molecular markers can predict the response to irinotecan-based systemic treatment and are associated with QoL and OS;
2. To validate the safety of *UGT1A1* genotype-guided dose adaptation (70% dose intensity) in UGT1A1 PMs;
3. To assess whether high bacterial GUS enzyme activity is associated with irinotecan-induced GI toxicity, decreased QoL and OS

The most important secondary objectives of this study are:

1. To investigate the dynamics in GUS activity and overall gut microbiota composition, diversity and functional capacity during irinotecan-based systemic treatment;
2. To assess associations between GUS activity and gut microbiota composition, diversity and functional capacity,
3. To measure fecal levels of SN-38 and SN-38-G and to determine its association with gut microbiota composition, bacterial GUS activity, *UGT1A1* genotype, treatment response and toxicity;
4. To correlate tumor molecular profile, GUS activity and other clinical characteristics (e.g. physical performance and nutritional status) during irinotecan-based systemic treatment.

### Study design

OPTIMA is a prospective, observational, multi-center cohort study conducted at the Maastricht University Medical Centre + (MUMC +, Maastricht), Catharina Hospital (Eindhoven), VieCuri Medical Center (Venlo), Amsterdam University Medical Center (AUMC, Amsterdam), Van Weel-Bethesda Hospital (Dirksland), Hospital Gelderse Vallei (Ede) and Rode Kruis Hospital (Beverwijk), all situated in the Netherlands. The study will be conducted according to the guidelines of the Declaration of Helsinki and Good Clinical Practice and is approved by the Medical Ethics Committee of azM/UM (METC 2022–3247)and the Institutional Review Board of MUMC + (RvB/301756/ReWi). This study has been registered in the ClinicalTrials.gov register (NCT05655780). All patients will sign written informed consent prior to enrollment.

#### Study population

Patients will be included via the oncologic outpatient clinics of the participating centers. Patients are eligible if they are ≥ 18 years of age, diagnosed with advanced CRC, and planned to start with irinotecan-based systemic treatment, if applicable in combination with anti-EGFR treatment. Additionally, patients must have a WHO performance status 0–2, minimal acceptable safety laboratory values at baseline, defined as: ANC of ≥ 1.5 × 109/L, platelet count of ≥ 100 × 109/L, serum bilirubin ≤ 1.5 × ULN, ALAT and ASAT ≤ 2.5 × ULN; in case of liver metastases ALAT and ASAT ≤ 5 × ULN, and renal function (eGFR) ≥ 50 ml/min or creatinine ≤ 1.5 × ULN.

Exclusion criteria are: microsatellite instability (MSI) or deficient MMR proteins, being pregnant or nursing, presence of an ileostomy, Asian ethnicity, other systemic treatment within < 1 month before inclusion, therapeutic antibiotic use within < 3 months before inclusion, abdominal radiotherapy within < 2 weeks before inclusion, being physically or mentally incapable or incompetent, more than 25% irinotecan dose reduction at the start of treatment (dose reductions during treatment are allowed), with exception of dose reduction in UGT1A1 PMs.

Patients can be treated with combination chemotherapy (FOLFOXIRI/FOLFIRI) or irinotecan monotherapy and will follow the regular treatment regimens, according to the Dutch guidelines (Table 1).

**Table 1:** Overview of irinotecan-based treatment regimens

		**Compounds**	**Number of weeks per cycle**	**Possible addition of bevacizumab?**	**Possible addition of anti-EGFR?**
Treatment	FOLFOXIRI	Irinotecan 5-FU Folinic acid Oxaliplatin	2	yes	no
	FOLFIRI	Irinotecan 5-FU Folinic acid	2	yes	yes
	Monotherapy	Irinotecan	3	no	no

#### Sampling time points

The sampling period encompasses the first six cycles of irinotecan-based systemic treatment. Before treatment initiation, patients will collect the first (baseline) fecal sample and fill out baseline questionnaires. Additionally, routine computed tomography (CT) or magnetic resonance imaging (MRI) scan as well as *UGT1A1* genotyping will be conducted. Afterwards, patients will enter the regular treatment cycles of irinotecan-based systemic treatment. During the first six cycles, they will regularly collect fecal samples and complete follow-up questionnaires (Table 2). The exact number of weeks between sampling time points might vary, depending on the treatment regimens and potential treatment delays. If available, archived tumor tissue will be used for molecular tumor profiling.

**Table 2:** Sampling timepoints of the OPTIMA study

		**Inclusion**	**Baseline**	**Cycle 1**	**Cycle 2**	**Cycle 3**	**Cycle 4**	**Cycle 5**	**Cycle 6**
Regular treatment	Hospital visit	X	X	X	X	X	X	X	X
	Archived tumor tissue		X						
	CT/MRI		X				X		
	Blood sample for UGT1A1		X						
Study related	Questionnaires		T1				T5		T6
	Fecal sample		T1	T2	T3	T4	T5		T6

### Sample collection and analysis

#### Tumor molecular profiling

If archived tumor samples are available from prior surgery or biopsy, the molecular profile of the tumor will be quantified using RNA sequencing for CMS classification and a DNA panel for mutational characterization. Therefore, a workflow will be generated using DNA/RNA from formalin-fixed paraffin-embedded (FFPE) tissues. These samples will be analyzed using RNA sequencing and the TruSight Oncology 500 (TSO500) assay, utilizing the Illumina NovaSeq6000 platform. The CMS classification of the tumor will be characterized based on a recently established FFPE CMS classification algorithm [19]. The TSO500 targets somatic variants over 523 relevant cancer genes.

#### UGT1A1 genotyping in blood samples and adaptation of irinotecan dose intensity

Before treatment initiation, the *UGT1A1* genotype of the patient will be assessed in blood using Polymerase Chain Reaction (PCR). Patients will be genotyped for UGT1A1*28 (TA repeat; rs3064744; NC_000002.12:g.233760235TA[5_11]). DNA for genotyping will be isolated from 200µl of whole EDTA blood obtained prior to start of therapy. Genotyping will be conducted using validated real-time PCR reactions with appropriate wild type, heterozygous and homozygous controls in every run. Genotypes will be converted to phenotypes in the following manner: homozygous carriers of UGT1A1*28 are defined as UGT1A1 PMs, heterozygous carriers are considered UGT1A1 intermediate metabolizers (IMs) and *UGT1A1* wild type individuals are considered UGT1A1 normal metabolizers (NMs). In UGT1A1 PMs the irinotecan starting dose will be reduced by 30% and further individualized in subsequent cycles of treatment based on ANC and clinical tolerance. The starting dose in UGT1A1 NMs and IMs will be left unchanged. Further dose modifications or treatment delays will be applied according to standard of care protocols and guidelines.

#### Quantification of bacterial GUS activity and gut microbiota composition in fecal samples

Fecal samples will be collected at home using a patient-friendly, and easy-to-use collection kit. At each time point, patients are asked to fill two fecal collection tubes with spoons attached to the lid (Sarstedt, Nümbrecht, Germany) and to immediately store samples in a freezer at home, until their next hospital visit. Cooled transporter container (Sarstedt, Nümbrecht, Germany) will be provided to the patients to ensure that the samples remain frozen during the transport to the hospital. Upon arrival in the hospital, samples will be temporarily stored at −20°C and at −80°C for long-term storage.

GUS activity in these samples will be quantified using an enzyme activity assay, which is based on the assay as described by Jariwala et al. and Bhatt et al. [15, 18] and which is currently under validation in our laboratories for the use with human fecal samples. In short, this assay consists of three steps: firstly, a pre-processing to remove unwanted debris, secondly a bicinchoninic acid (BCA) assay (Pierce™ BCA Protein Assay Kit, Thermo Fisher Scientific, USA) to determine the total protein concentration in the fecal lysate, and thirdly a fluorescence-based enzyme activity assay quantifying SN-38G-specific GUS activity. For the enzyme activity assay, fluorescent SN-38G is added to the fecal lysate and conversion to SN-38 during a period of one hour is quantified using a SpectraMax iD3 multi-mode microplate reader (Molecular Devices, San Jose, CA, USA). Subsequently, an online algorithm-based calculation tool (developed by the University of North Carolina, USA) will be used to calculate SN-38 reactivation rate as concentration over time (nM/min). Fecal levels of SN-38 and SN38-G will also be measured directly in fecal samples by means of a liquid chromatography-mass spectrometry (LC–MS/MS) assay.

Gut microbiota composition, diversity and functional capacity will be analyzed by means of whole metagenomic shotgun sequencing (WMGS). Fecal samples collected at baseline (T1) as well as during the first (T2) and fourth (T5) cycle of the irinotecan-based systemic treatment will be used for this analysis. From these samples, 150-250mg of feces will be weighted and metagenomic DNA will be isolated using the QIAamp DNA Mini kit (Qiagen, the Netherlands). In short, this procedure will consist of bead-beating with zirconia beads and the Fastprep™ instrument (MP Biomedicals, USA), followed by column-based DNA purification. Library preparation will be performed with the Illumina DNA Prep kit (M) (Illumina, USA). Subsequently, WMGS will be performed on a NovaSeq 6000 Illumina platform (sequencing depth > 10 Gb/sample, loading 300–600 pM). Filtered reads will be assembled into metagenome assembled genomes (MAGs) as well mapped to the HUManN3 database [20] to assed the functional capacity of the gut microbiomes of the samples. The MAG assembly, including a taxonomic and functional annotation of the MAGs will be done with the publicly available nf-core/mag pipeline [21, 22]. For the read-level annotation both a taxonomic as well as functional assigned using the HUMaN3 package [20] will be used to assess the general functionalities for the microbial communities. The datasets will be combined to obtain the functional potential and taxonomic composition of the gut microbiomes.

### Clinical data collection and management

#### Evaluation of tumor response

Tumor response will be evaluated based on standard care CT or MRI scans conducted at baseline (T1) and after the fourth cycle (T5) of irinotecan-based systemic therapy. Based on the percentage of change of target lesions, tumor response will be scored according to the Response Evaluation Criteria in Solid Tumors (RECIST, version 1.1 [23]) and will be classified as progressive disease, stable disease, partial response or complete response.

#### Questionnaires

During the study period, patients are asked to fill out questionnaires at T1, T5, and T6 (Table 1). Amongst others, the questionnaires encompass questions concerning medical history, comorbidities, dietary patterns, the use of pre-/probiotics, the use of antibiotics and other co-medication, smoking, and alcohol use. Furthermore, patients are asked to score their physical functioning (based on the Karnofsky Performance Score, KPS), the risk of malnutrition (based on the MUST), as well as the occurrence of systemic and gastrointestinal toxicity (based on the CTCAE criteria, version 5.0 [24]). Furthermore, the European Organization for Research and Treatment of Cancer (EORTC) QLQ-C30 and the EQ-5D-5L questionnaires are used to quantify QoL.

#### Additional information

If available, blood inflammatory markers (e.g. leukocyte counts) will be retrieved from the medical records of participating patients. Furthermore, clinical information derived from the questionnaire will be completed with relevant data from the medical records.

#### Data management

All clinical data collected will be collected in an electronic case report form (eCRF) using the clinical data management platform CASTOR. Personal data will be handled with strict care and following Good Clinical Practice (GCP) guidelines. Collected data and samples will be coded with a pseudonymized study code, the key linking study code to patient data will only be accessible to the primary investigator as well as the coordinating researcher(s). Conform current guidelines, all data and samples will be stored for a maximum of 15 years and can be used for any future studies in line with the current research.

### Statistical and bioinformatic analyses

#### Sample size calculation

The sample size calculation was based on having sufficient precision to estimate sensitivity and specificity of the GUS activity assay to predict late-onset GI toxicity. Literature shows that about 82% of patients treated with irinotecan experience diarrhea (all grades) [25], while approximately 18% develop grade 3–4 diarrhea [5, 6]. For estimation of sensitivity, we determined a maximum half-width of the 95% confidence interval (CI) of 17%, requiring a minimum of 22 patients with grade 3–4 diarrhea. To achieve this precision, a total of 84 patients need to be included, considering an estimated toxicity rate ranging from 16 to 22%. Out of these 84 patients, 62 individuals would not experience grade 3–4 diarrhea, allowing for the estimation of specificity with a 95% CI half-width of around 10%. Hence, the total number of patients required remains at 84. Considering an anticipated 20% drop-out rate, because of the advanced setting, 104 patients need to be included in the study.

#### Statistical and bioinformatic analysis of main outcome parameters

The association between CMS subtypes 1–4 and treatment response (categorized as progressive disease, stable disease, partial response and complete response) will be analyzed using chi-squared tests. Survival analysis within the different CMS subtypes will be done by Kaplan Meier curves.

To validate the safety of the 70% irinotecan starting dose intensity, toxicity in UGT1A1 PMs will be compared to historical control patients, i.e. cohorts of patients described in literature that are homozygous polymorphic for UGT1A1*28 and were treated with full dose therapy. In addition, toxicity in UGT1A1 PMs will be compared to UGT1A1 non-PMs treated with full dose in the current study, under the assumption that these groups experience comparable degrees of toxicity.

Correlations between GUS activity and the degree of experienced late-onset GI toxicity during irinotecan-based treatment will be analyzed by means of the Spearman correlation coefficient. Furthermore, we will investigate whether GUS activity is different between patients without diarrhea vs. grade 1–2 diarrhea vs. grade 3–4 diarrhea by means of ANOVA or the Kruskal Wallis test, depending on whether the data are normally distributed or not. Longitudinal changes in GUS activity during irinotecan-based treatment will be analyzed using repeated-measures ANOVA or the Friedman test.

Bioinformatic analysis of the predicted GUS functionality derived from metagenomics sequencing will be conducted. A Hidden Markov Model (HMM)-profile will be made per orthologous group with predicted GUS functionality, based on a reference dataset of publicly available GUS protein sequences and the results of Pollet et al. [12, 26]. The HMM-profiles will be used to identify all predicted proteins with a potential GUS functionality per fecal sample. These predicted proteins will be used to create a non-redundant microbial GUS protein catalogue.

Changes in GUS activity and overall gut microbiota composition, diversity and potential GUS capacity during irinotecan-based systemic treatment will be assessed by redundancy analysis (RDA) [27] and ANOVA [28]. These data will be used to examine the microbial GUS variation and origin per fecal community in order to link the microbial composition and GUS functional potential to the measured GUS activity.

In addition, the main outcome parameters will be correlated to fecal levels of SN-38 and SN-38G, as well as other clinical variables of interest, as assessed by the questionnaires.

## Discussion

To the best of our knowledge, the OPTIMA study will be the first study to evaluate tumor molecular profiling, *UGT1A1* genotype and GUS activity as potential biomarkers for efficacy and toxicity of irinotecan-based systemic treatment in patients with advanced CRC. By combining these three biomarkers into one study, we will provide pivotal new insights for clinicians to make well-considered choices concerning for instance the initiation, dose adjustments or the need for microbiota modulation during irinotecan-based systemic treatment in this patient population.

The tumor molecular profiling will provide new insights into which patients are expected to benefit from irinotecan-based systemic treatment. As outlined in the introduction, irinotecan-based systemic treatment is probably not the optimal choice in all CMS subtypes. Validation of these previous findings in the current cohort will significantly strengthen the rationale for a more personalized treatment selection based on the tumor molecular profile.

Furthermore, OPTIMA will further evaluate the use of *UGT1A1* genotype and subsequent dose adjustments in clinical practice. If a 70% dose intensity in UGT1A1 PMs leads to similar incidences of severe toxicity as compared to UGT1A1 NMs and IMs treated with the full dose, this further supports the validity and utility of this biomarker.

In addition, new knowledge on the role of bacterial GUS in a clinical setting, will enhance the detection of patients who are expected to experience high GI toxicity before start of the treatment. This opens the opportunity to modulate GUS activity in these patients by targeted gut microbiota modulation.

However, there are some limitations inherent to the current study. Due to the inclusion of patients receiving different treatment regimens, as well as potential treatment delays in individual patients, the time between two consecutive sampling time points might differ between patients. Additionally, we only ask patients to report overall dietary patterns and do not collect detailed data on dietary intake, which might influence particularly the gut microbiota parameters. Lastly, due to the advanced setting, patients might have distinct and complex medical histories, co-morbidities and co-medication which could influence the outcomes.

On the other hand, the unique strengths of the OPTIMA study are the multidisciplinary and multi-center character, the combination of three different biomarkers as well as the longitudinal design with repeated sampling per patient. In addition, we include baseline sampling before treatment initiation, which enables us to investigate treatment-related effects. The comprehensive collection of biological specimens as well as clinical data is a further advantage of the current study.

To conclude, results obtained in the context of the OPTIMA study will greatly contribute to the optimization of efficacy and the reduction of toxicity of irinotecan-based systemic treatment, thereby significantly improving QoL of patients. In view of the fact, that the maintenance of QoL is of particular relevance in the palliative setting, the OPTIMA study will be of great benefit for patients as well as their caregivers.

## Data Availability

No datasets were generated or analysed during the current study.

## Abbreviations

ALAT: Alanine aminotransferase
ANC: Absolute neutrophil count
ASAT: Aspartate aminotransferase
BCA: Bicinchoninic acid
CI: Confidence interval
CMS1-4: Consensus molecular subtypes 1–4
CRC: Colorectal cancer
CT: Computed tomography
CTCAE: Common Terminology Criteria for Adverse Events
EORTC: European Organization for Research and Treatment of Cancer
eCRF: Electronic case report form
FFPE: Formalin-fixed paraffin-embedded
GI: Gastrointestinal
GCP: Good Clinical Practice
GUS: Bacterial enzyme β-glucuronidase
HMM: Hidden Markov Model
IMs: Intermediate metabolizers
KPS: Karnofsky Performance Status
LC–MS/MS: Liquid chromatography coupled to tandem mass spectrometry
MAGs: Metagenome assembled genomes
MRI: Magnetic resonance imaging
MSI: Microsatellite instability
MUMC +: Maastricht University Medical Centre +
MUST: Malnutrition Universal Screening Tool
NMs: Normal metabolizers
OS: Overall survival
PCR: Polymerase Chain Reaction
QoL: Quality of life
RDA: Redundancy analysis
RECIST: Response Evaluation Criteria in Solid Tumors
TSO500: TruSight Oncology 500
UGT1A1: UDP Glucuronosyltransferase Family 1 Member 1
ULN: Upper limit of normal
WMGS: Whole metagenome shotgun sequencing
